# Red Angico Gum: A Nontoxic Polysaccharide with Promising Antitumor and Antiedematogenic Activity

**DOI:** 10.1021/acsomega.5c09191

**Published:** 2025-11-13

**Authors:** Dakson Douglas Araújo, Rayran Walter Ramos de Sousa, Débora Caroline do Nascimento Rodrigues, Ingredy Lopes dos Santos, Paulo Michel Pinheiro Ferreira, Antonielly Campinho dos Reis, Maria Luísa Lima Barreto do Nascimento, João Marcelo de Castro e Sousa, Fábio de Oliveira Silva Ribeiro, Durcilene Alves da Silva, Irisvan Silva Ribeiro, Venicios Gonçalves Sombra, Regina Célia Monteiro de Paula, Nayze Lucena Sangreman Aldeman, José Delano Barreto Marinho Filho, Ana Jérsia Araújo

**Affiliations:** 1 Laboratório de Cultura de Células do Delta (LCCDelta), 603028 Universidade Federal do Delta do Parnaíba, Parnaíba, PI 64202-020, Brazil; 2 Laboratório de Cancerologia Experimental (LabCancer), Departamento de Biofísica e Fisiologia, 67823 Universidade Federal do Piauí, Teresina, PI 64049-550, Brazil; 3 Laboratório de Genética Toxicológica (LAPGENIC), Departamento de Bioquímica e Farmacologia, Universidade Federal do Piauí, Teresina, Teresina, PI 64049-550, Brazil; 4 Núcleo de Pesquisa em Biodiversidade e Biotecnologia (BIOTEC), 603028 Universidade Federal do Delta do Parnaíba, Parnaíba, PI 64202-020, Brazil; 5 Laboratório de Polímeros (LabPol), 28121 Universidade Federal do Ceará, Fortaleza, CE 60455-760, Brazil; 6 Curso de Medicina, 603028 Universidade Federal do Delta do Parnaíba, Parnaíba, PI 64202-020, Brazil; 7 Faculdade de Ciências Humanas, Exatas e da Saúde do Piauí, Instituto de Educação Superior do Vale do Parnaíba, Parnaíba, PI 64212-790, Brazil

## Abstract

Polysaccharides are complex carbohydrates that play essential roles in several biological processes. The aim of this study was to isolate, characterize, and evaluate the cytotoxic, antitumor, and antiedematogenic potential *in vitro* and *in vivo* of red angico gum (RAG), a polysaccharide isolated from *Anadenanthera colubrina* var. *cebil* (Griseb.) Altschul. To confirm the chemical characteristics of RAG, nuclear magnetic resonance, size-exclusion chromatography, high-performance liquid chromatography analysis, and determination of uronic acid content by potentiometric titration were performed. RAG is composed mainly of arabinose and galactose with smaller proportions of rhamnose and glucuronic acid. *In vitro* cytotoxicity was determined by MTT and Alamar blue assays. For *in vivo* assays, mice bearing a sarcoma 180 tumor and the carrageenan-induced paw edema model were used to evaluate the antitumor and antiedematogenic effects. Hematological, biochemical, histopathological, and mutagenic analyses were performed to observe signs of toxicity after RAG administration. RAG did not show cytotoxicity *in vitro*; in contrast, it showed a significant tumor inhibition *in vivo* of up to 46.5%. Notably, such effects were observed without causing systemic and tissue toxicity in animals. Furthermore, RAG administration significantly reduced paw edema. These findings suggest that this polysaccharide exhibits antitumor and antiedematogenic potential in animal models, without showing signs of toxicity.

## Graphical abstract

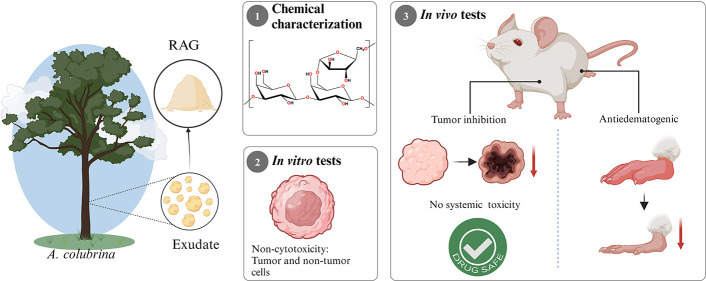

## 1 Introduction

Cancer is a serious public health issue and is associated with one of the leading causes of death worldwide. Incidence and mortality statistics have gradually increased each year.^1,2^ In 2022, approximately 20 million new cancer cases were estimated globally, reflecting the growing global impact of this disease. Demographic-based projections indicate that by 2050, the annual number of new diagnoses could reach around 35 million.^3^ Among the different types of cancer, sarcomas are a heterogeneous group of cancers originating from mesenchymal tissues and can be classified into soft tissue and bone sarcomas.^4^ Sarcomas are relatively more common in children and young adults and are broadly classified into more than 70 subtypes occurring in muscles, cartilage, blood vessels, nerves, and adipose tissues.^1^

The main treatments used in cancer therapy include surgery, radiotherapy, and chemotherapy, which can be applied individually or in combination, depending on the characteristics of the tumor. Chemotherapy stands out for its effectiveness in destroying neoplastic cells; however, its use is often limited by high toxicity and severe adverse effects.^1^ Despite advances in the discovery of drugs that can be implemented in cancer therapy, prevention and control still represent a great challenge due to the unique characteristics of each type of cancer.^5^ The mechanisms of resistance by tumor cells, low efficacy for different types of tumors, low-selectivity drugs, and a wide spectrum of adverse effects caused by conventional therapy stand out as limitations in therapy.^1,6,7^ Therefore, the quest for discovering and identifying new alternative therapeutic agents that are effective and have fewer toxic effects is garnering increasing interest within the scientific community. This pursuit aims to find applications for this group of diseases.

Among the many compounds isolated from natural sources, polysaccharides are designated as a natural macromolecular polymer, which has no less than 10 monosaccharide units connected by glycosidic bonds and have various biological applications.^8,9^ Therefore, they have been gaining visibility due to their great variety, versatility, pharmacological properties, absence of or low toxicity, low cost, and biodegradability.^10,11^ Arabinogalactans are an important class of polysaccharides primarily composed of galactose and arabinose and are classified into type I and II. Type I arabinogalactans are usually bound to the rhamnogalacturonan I backbone of pectin; type II are usually found as carbohydrate moieties of arabinogalactan proteins. These compounds are widely distributed throughout the plant kingdom^12,13^ and as reported in the literature exhibit notable antitumor and immunomodulatory,^10,14–16^ anti-inflammatory,^17^ and antioxidant^18^ properties.

*Anadenanthera colubrina* (A. colubrina) var. *cebil* (Griseb.) Altschul, popularly known as red angico, is a plant belonging to the Fabaceae family,^19,20^ considered one of the most relevant medicinal plants present in the Brazilian Caatinga and Cerrado biomes.^21,22^ Several studies have demonstrated different pharmacological effects of products derived from *A. colubrina*, in particular extracts and fractions of leaves and roots that include anti-inflammatory,^23–25^ healing,^26^ antimicrobial,^22,27,28^ antinociceptive,^25^ antioxidant,^23,29–31^ protective effect of the laryngeal and esophageal mucosa,^32^ and antiproliferative properties.^31,33^

The red angico gum (RAG) isolated from the exudate produced in the trunk of *A. colubrina* has characteristics of a heteropolysaccharide composed of 67–68% arabinose, 24% galactose, 2% rhamnose, and 6–8% glucuronic acid, designated as an arabinogalactan type II.^34–37^ RAG analysis showed that the isolated polysaccharide presented a →3)-β-d-galactopyranose­(1→ main chain and side chains of →6)-β-d-galactopyranose­(1→, →3,6)-β-d-galactopyranose­(1→, and →3)-α-l-arabinofuranose-(1→ and terminal groups of α-rhamnose, β-glucuronic acid, α-arabinofuranose, β-arabinopyranose, and α,β-galactose.^36,37^ In addition, RAG showed antidiarrheal effects in an *in vivo* model by increasing Na^+^/K^+^-ATPase activity, reducing gastrointestinal transit through inhibiting smooth muscle contractions and interaction with GM1 receptors.^21^

RAG demonstrated no toxicity in both *in vitro* and *in vivo* studies conducted on *Galleria mellonella* larvae, as well as human embryonic cell lines, murine fibroblasts, and human erythrocytes.^11,38^ Furthermore, a single oral dose of 2000 mg/kg of RAG in acute treatment showed no toxic effects in the studied model.^21^ Based on these findings, we propose that RAG exhibits antitumor and antiedematogenic effects with minimal associated toxicity.

This study aims to investigate the antitumor potential and toxicological profile of RAG *in vivo* using an experimental mouse model transplanted with sarcoma 180 (S180) tumor cells. Additionally, we evaluated the antiedematogenic effect of RAG in a carrageenan-induced paw edema mouse model.

## 2 Materials and Methods

### 2.1 Drugs and Chemicals

Fetal bovine serum (FBS) and Dulbecco MEM (DMEM) and Roswell Park Memorial Institute Medium 1640 (RPMI) culture media were obtained from Gibco Industries, Inc. (Grand Island, NY, USA). MTT, resaruzin, penicillin and streptomycin, dimethyl sulfoxide (DMSO), λ-carrageenan (type IV Lambda), and 5-fluorouracil (5-FU) were obtained from Sigma Chemical Company (St. Louis, Mo, USA). 0.25% trypsin was purchased from Thermo Scientific HyClone (Logan, UT, USA). Indomethacin was purchased from Vetec Química (São Paulo, Brazil). All other chemical products used were of analytical grade, obtained from accredited companies and used according to each manufacturer.

### 2.2 Isolation and Characterization of RAG

#### 2.2.1 Plant Exudate

Crude exudate samples were collected from the trunks of the native red angico (*A. colubrina*) in the municipality of Simplício Mendes, Piauí, Brazil (latitude, S–7.85388994216919; longitude, W–41.9103012084961). The material collected (voucher number 3618) was deposited at the Herbarium of the Parnaíba Delta (HDELTA) from Universidade Federal do Delta do Parnaíba, Parnaíba, Piauí, Brazil. The registry in SisGen (Sistema Nacional de Gestão do Patrimônio Genético e do Conhecimento Tradicional Associado–National System of Management of Genetic Heritage and Associated Traditional Knowledge: #A77A292) is in accordance with Brazilian Federal Law No. 13123/2015 about access to national biodiversity.

#### 2.2.2 Isolation and Purification of RAG

RAG was isolated and purified according to the protocol described by Ribeiro et al.^38^ Briefly, 10% (w/v) exudate solution was prepared in distilled water and stirred at 25 °C for 24 h. After this time, it was filtered through a sintered glass funnel with porosity 4, under vacuum, and the pH was adjusted to 7 (NaOH, 1 mol/L). 2 g of sodium chloride (NaCl) was added to the filtered solution, and it was left stirring for 30 min. Precipitation was performed in 99.5% ethyl alcohol in a 3:1 (v/v) ratio and allowed to stand at 25 °C for 24 h. The precipitated gum was washed with ethyl alcohol and acetone and then dried under hot air.

#### 2.2.3 Nuclear Magnetic Resonance (NMR)

Proton and carbon nuclear magnetic resonance analyses (^1^H NMR, ^13^C NMR, proton broad-band decoupling, gated decoupling, and ^1^H–^13^C HSQC) were performed using the Bruker Avance DRX 500 model equipment, with temperature control set at 40 °C. Sodium 2,2-dimethyl-2-silapentane-5-sulfonate (DSS) was used as an internal standard, and the sample (50 mg) was prepared in deuterated water (D_2_O).

#### 2.2.4 Size-Exclusion Chromatography (SEC)

The molecular weight distribution of RAG was estimated by SEC using a Shimadzu system equipped with an LC-20AD pump and coupled to a refractive index detector (RID-10A). For the analysis, a PolySep-GFC-P Linear column (300 × 7.8 mm) and NaNO_3_ (aq) (0.1 mol/L) were employed as the eluent. Measurements were conducted at 30 °C, with a flow rate of 1 mL/min and a sample (0.1 mg/mL) injection volume of 50 μL. The molecular weight was calculated using a standard pullulan curve (eq 1).

$$\log M=-1.23456\times V_{e}+14.88194R^{2}=0.996$$ 1

where *V*_e_ is the elution volume.

#### 2.2.5 Hydrolysis and High-Performance Liquid Chromatography (HPLC)

The hydrolysis of the polysaccharide RAG was carried out using trifluoroacetic acid (TFA). RAG (10 mg) was dispersed in distilled water (1 mL) and kept under agitation for 24 h. Then, TFA (1 mL, 4 mol/L) was added, and the solution was transferred to a sealed tube, which was heated at 100 °C in a glycerin bath for 2.5 h. Subsequently, the hydrolyzed material was transferred to a glass vial, and excess acid was evaporated under a stream of nitrogen gas. The hydrolyzed material was then washed with methanol (5 times), and the methanol was removed at 40 °C using a rotary evaporator IKA Model HB ECO S099. The monosaccharide composition of RAG was estimated by HPLC using a Shimadzu system equipped with an LC-20AD pump and coupled to a refractive index detector (RID-10A). For the analysis, a Rezex ROA-Organic Acid H^+^ (8%) column (300 × 7.8 mm) and H_2_SO_4_ (aq) (8 mmol/L) were used as the eluent. Identification was performed using monosaccharide standards.

#### 2.2.6 Ash Content

Ash was determined using thermogravimetric analysis. The thermogram was obtained in a Shimadzu DTG-60H, under an oxidative atmosphere (synthetic air, 40 mL/min), at a heating rate of 10 °C/min, from 25 to 1000 °C. The residue was determined at 600 °C.

#### 2.2.7 Determination of Acid Content by Potentiometric Titration

The content of acid groups in RAG was estimated by potentiometric acid/base titration. RAG (0.1 g) was dispersed in distilled water (25 mL), and the dispersion was eluted with ultrapure water (75 mL) through an ion exchange column (Amberlite IR-120) to protonate the acid groups of RAG. Then, the sample was frozen and lyophilized. Subsequently, the protonated RAG was dispersed in ultrapure water and titrated with standardized sodium hydroxide.^39^

#### 2.2.8 Elemental Analyses

The nitrogen content was determined using a PerkinElmer 2400 series CHNS analyzer with a thermal conductivity detector. The conversion factor for %N in protein used was 5.87.^40^

### 2.3 Animals

Sixty-four female mice (*Mus musculus*, Swiss strain) 6 to 8 weeks old and weighing 25–35 g were obtained from the facilities of Federal University of Piauí, Teresina, Brazil. All animals were kept under standard conditions of light (12:12 h light/dark cycle) and temperature (23 ± 1 °C) and were housed with free access to commercial rodent stock diet (Nutrilabor, Campinas, Brazil) and potable water *ad libitum*. After the tests, mice were anesthetized or euthanized with xylazine (4.5 or 30 mg/kg) and ketamine (90 or 300 mg/kg) by an intraperitoneal route (i.p.), respectively. All protocols established with the use of animals were submitted and approved by the Ethics Committee on Animal Experimentation of UFPI (CEUA No. 555/19) and followed Brazilian (Brazilian Society of Science in Laboratory Animals-SBCAL) and international rules (Directive 2010/63/EU) on the care and use of animals in experiments.

### 2.4 *In Vitro* Cytotoxicity

#### 2.4.1 Cell Culture

The tumor cell lines used were CT26.WT (murine colorectal carcinoma), HCT116 (human colorectal carcinoma), 4T1 (murine mammary carcinoma), and S180 (murine sarcoma). The nontumor cell lines were L929 and 3T3 (murine fibroblasts). All cell lines were handled in a vertical laminar flow chamber (Pachane) and kept in an incubator at 37 °C and an atmosphere with 5% CO_2_ (Thermo Scientific Series 8000 WJ). The cell lines were grown in culture flasks (25 cm^2^, 50 mL volume or 75 cm^2^, 250 mL volume) containing DMEM or RPMI culture medium supplemented with 10% FBS and 1% antibiotic (to a final concentration of 100 U/mL penicillin and 100 μg/mL streptomycin).

Sarcoma 180 cells were maintained in the intraperitoneal region of Swiss mice as ascitic tumors. Maintenances were performed every 10 days where donor mice were euthanized with an overdose of anesthetics; ascitic fluids containing cells were collected and transplanted into new mice intraperitoneally under aseptic conditions, ensuring the maintenance of the tumor.

#### 2.4.2 MTT Assay

Colorimetric assays were performed to evaluate the cytotoxicity of RAG against tumor and nontumor cells through the reduction of 3-(4,5-dimethyl-2-thiazole)-2,5-diphenyl-tetrazolium bromide (MTT) in metabolically active cells.^41^ Cells were distributed in 96-well plates at a density according to the doubling time of each lineage. After this, cells were treated for 69 h with serial concentrations of RAG, ranging from 15.6 to 1000 μg/mL. The MTT solution (0.5 mg/mL) was added, and the plates were reincubated for a further 3 h. Finally, the MTT formazan product was dissolved in DMSO and the absorbances were obtained using a plate spectrophotometer (Beckman Coulter, Inc., model DTX-880), using Multimode Detection Software (Beckman Coulter, Inc.) at a wavelength of 595 nm. Absorbance values were transformed into percent inhibition, based on the mean absorbance of the negative control.

#### 2.4.3 Alamar Blue

Initially, mice with 9 to 10 days of ascitic tumors were euthanized and a suspension of the S180 cell line was collected from the intraperitoneal cavity under aseptic conditions.^42,43^ A cell suspension was centrifuged at 2000 rpm for 5 min to obtain a pellet, which was washed three times with sterile RPMI medium. The cell concentration was adjusted to 5 × 10^5^ cells/mL in RPMI 1640 medium supplemented with 10% FBS, 100 U/mL penicillin, and 100 μg/mL streptomycin. Cells were plated in 96-well plates with RAG concentrations ranging from 15.6 to 1000 μg/mL and incubated at 37 °C in a 5% CO_2_ atmosphere (Shel Lab CO_2_ incubator, USA).

Cell proliferation was assessed after 72 h of exposure. At 68 h of incubation, 10 μL of Alamar Blue stock solution of resaruzin (0.156 mg/mL, Alamar Blue, Sigma-Aldrich, USA) was added to each well and left for an extra 4 h. Cell proliferation was determined by fluorescence intensity using a multiplate reader (GloMax Discover Microplate Reader) with excitation (520 nm) and emission (580–640 nm). The antiproliferative effect was expressed as a percentage of the control.^43^

### 2.5 Antitumor Potential and *In Vivo* Toxicological Parameters

#### 2.5.1 Experimental Design

Ten-day-old S180 ascite tumor cells were removed from the peritoneal cavity, counted, and subcutaneously implanted into the right hind axillary region of healthy Swiss animals (6 × 10^6^ cells/0.5 mL).^43,44^ On the next day, animals were randomly divided into four groups (*n* = 8 animals/group) and the substance dissolved in saline 0.9% was intraperitoneally injected for 10 consecutive days (50 and 100 mg/kg/day). Negative and positive controls received saline (0.9%) and 5-FU (15 mg/kg/day, i.p.), respectively. On the 11th day, the animals were anesthetized with ketamine (90 mg/kg) plus xylazine (4.5 mg/kg) for blood collection from each animal via cardiac puncture.^45^

Next, the animals were euthanized, and organs (spleen, heart, liver, and kidneys) and tumors were collected by surgical techniques, weighed, and stored in 10% buffered formalin. On the next day, they were preserved in 70% alcohol for further histopathological analysis. Tumor inhibition (TI) was calculated as follows: TI (%) = [(*A* – *B*)/*A*] × 100, where *A* is the mean weight of tumors in the negative control group and *B* is the mean tumor weight in treated animals.

#### 2.5.2 Hematological Analyses

For hematological analysis, an aliquot (∼500 μL) of blood was collected from each animal into sterile tubes containing ethylenediaminetetraacetic acid (EDTA). Various hematological parameters, including erythrocytes, leukocytes, and platelets, were evaluated using an automated analyzer (XS-1000i hematology analyzer, Sysmex).

#### 2.5.3 Biochemical Analyses

Biochemical analyses were performed on serum samples obtained by centrifuging whole blood without an anticoagulant at 1500 rpm for 10 min. The biochemical parameters evaluated included aspartate aminotransferase (AST), alanine aminotransferase (ALT), creatinine, alkaline phosphatase, and gamma-glutamyl transferase (GGT), following the manufacturers’ instructions. All analyses were conducted using a multiwell spectrophotometer (GloMax Discover Microplate Reader).

#### 2.5.4 Micronucleus Assay

Micronucleus tests (MN) using bone marrow cells were performed according to Krishna and Hayashi.^46^ To determine the frequency of micronucleated polychromatic erythrocytes (EPCMN), which characterizes the possible mutagenic effects, a total of 1000 polychromatic erythrocytes (EPCMN) were evaluated per animal (8000 cells per group). To determine the cytotoxicity, 500 erythrocytes from the bone marrow (EPC + ENC) per animal were counted (4000 cells/group). The cytotoxicity ratio for the bone marrow was calculated by the ratio EPC/ENC. Images were acquired using a plan achromatic infinity optical biological microscope (Bioptika brand model B605) with a 10-megapixel color CMOS digital camera (magnification, 1000×; software IS Capture 2.5, version 2.5.1547.4007).

#### 2.5.5 Relative Index of Organs and Histopathological Analyses

Collected organs such as the spleen, heart, liver, and kidneys of each animal were weighed to assess the relative index of the organs based on the formula: relative index (RI) = (organ weight/body weight) × 100. The histopathological analysis was performed according to Barros et al.^10^ The excised tissues were removed from 70% alcohol, embedded in paraffin, and sectioned using a microtome. Paraffin sections containing the tissue were fixed on slides and stained with hematoxylin and eosin. Tissue-specific toxicity changes were observed and photographed using an optical microscope.

### 2.6 Carrageenan-Induced Paw Edema

The health animals were randomly divided into four groups (*n* = 8 animals/group), and edema was induced by the administration of 50 μL of a carrageenan suspension (500 μg/paw) in vehicle solution to the right hind paw. The animals were pretreated intraperitoneally (i.p.) 30 min before carrageenan administration with RAG at doses of 50 and 100 mg/kg, indomethacin at 10 mg/kg (positive control), and vehicle (negative control). The paw volume was measured before (*V*_0_) and at 1, 2, 3, and 4 h after carrageenan treatment (*V*_t_) using a plethysmometer (Panlab, Barcelona, Spain). The pretreatment effect was calculated as a percentage of edema inhibition in relation to the paw volume of the vehicle group.^47,48^

### 2.7 Statistical Analysis

The half-inhibitory concentration (IC_50_) was determined by nonlinear regression (GraphPad Prism 9.0, Intuitive Software for Science, USA). Differences were evaluated by comparing data using one-way analysis of variance (ANOVA) followed by the Newman–Keuls test or Dunnett’s test (*p* < 0.05) using the GraphPad program. All *in vitro* studies were carried out in duplicate and represented independent biological evaluations.

## 3 Results and Discussion

### 3.1 Characterization of RAG

The elution volume at the peak of the SEC chromatogram (Figure 1A) was 8.00 mL, using a standard pullulan curve (eq 1), with a molar mass of RAG Mw = 1.0 × 10^5^ g/mol, which was estimated by SEC. The molar mass was lower than that obtained by Chaves et al.^49^ for the same species (1.89 × 10^5^ g/mol); however, this variation may be attributed to biological and environmental factors such as the age of the tree, geographic origin, and the season in which the exudate was collected, which are known to influence the physicochemical characteristics of plant polysaccharides. The HPLC chromatogram of hydrolyzed RAG confirmed the presence of galactose and arabinose (Figure 1B). Proximate analysis showed that isolated RAG contained 1.5% protein and 0.9% ash, and the purity of RAG was estimated to be 98.8% by difference.

**Figure 1:**
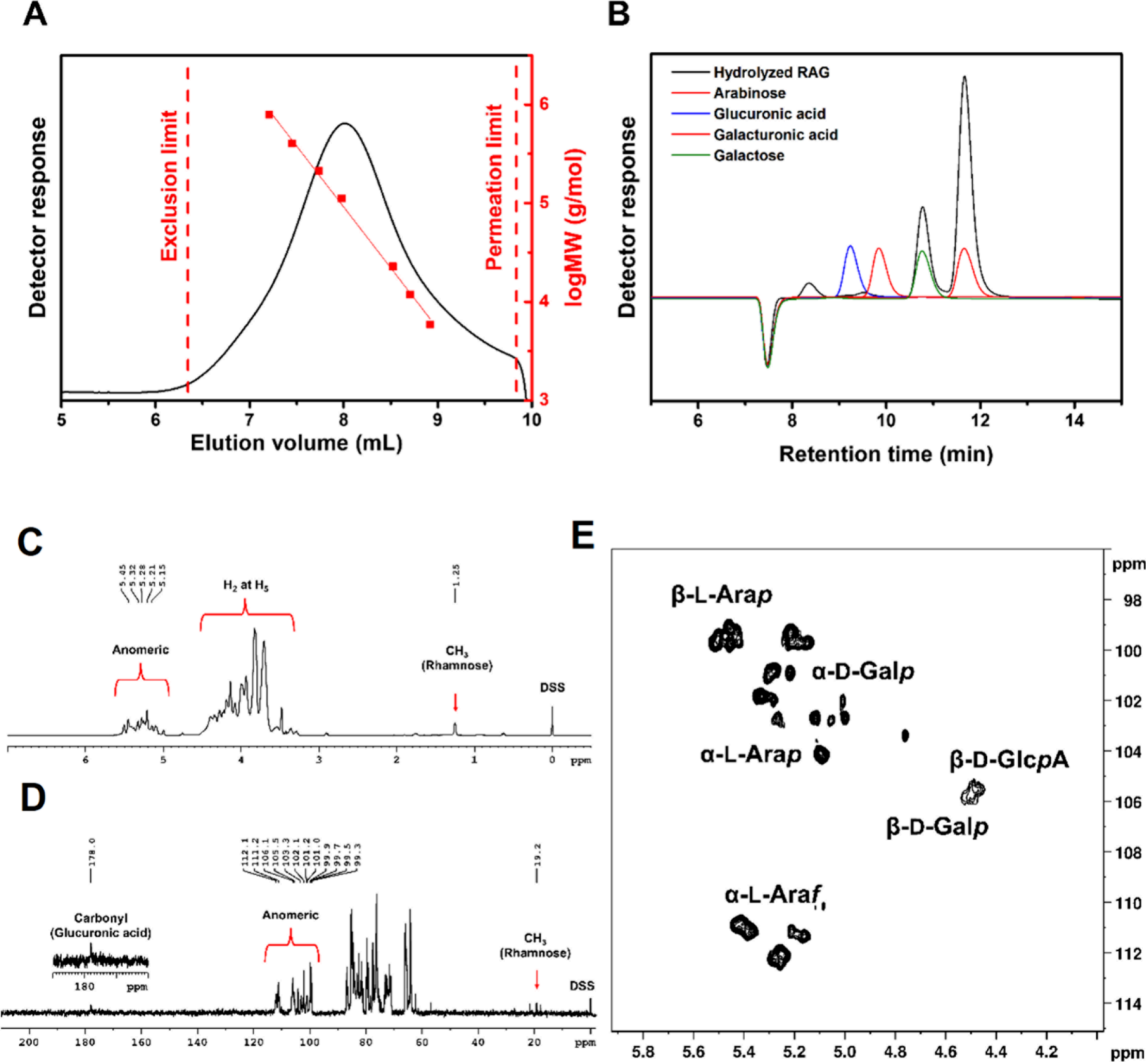
Characterization of RAG: SEC (A), HPLC (B), ^1^H NMR (C), ^13^C NMR (D), and ^1^H–^13^C HSQC (E).

The composition was assessed through a combination of techniques: HPLC, NMR, and potentiometric acid–base titration. The content of acid groups (5.5%) was estimated via potentiometric acid–base titration. The amount of rhamnose was calculated from ^1^H NMR, considering the signal of methyl (CH_3_) hydrogens of rhamnose, identified at 1.25 ppm, along with the anomeric signal (Figure 1C). The rhamnose content was found to be 1.5%. The amount of galactose (24.9%) and arabinose (68.1%) was estimated by HPLC analysis of hydrolyzed RAG; however, the presence of rhamnose sugar was not observed. Its absence can be attributed to its propensity for rapid hydrolysis (Figure 1B). The above-mentioned results confirm the structure previously reported by the target literature of the research.^35,36,38,49^

The RAG was characterized by NMR (Figure 1). The ^1^H NMR spectrum of RAG (Figure 1C) provides the characteristic chemical shifts (δ_DSS_) of the anomeric hydrogens of RAG between 5.5 and 5.0 ppm. The signals corresponding to the H_2_ to H_5_ hydrogens of the galactose, arabinose, and rhamnose units are observed overlapping between 4.4 and 3.3 ppm. The signal attributed to the methyl (CH_3_) hydrogens of rhamnose is identified at 1.25 ppm. The chemical shifts of the anomeric hydrogens of α-l-arabinofuranose (α-l-Ara*f*) at different positions in the polymer chain were observed at δ_DSS_ 5.43, 5.38, 5.26, and 5.15 ppm; those of β-l-arabinopyranose (β-l-Ara*p*) at δ_DSS_ 5.51, 5.46, 5.25, and 5.15 ppm; and that of α-l-arabinopyranose (α-l-Ara*p*) at δ_DSS_ 5.09 ppm. Anomeric hydrogens of β-d-galactopyranose (β-d-Gal*p*), β-d-glucuronic acid (β-d-GlcA), and α-d-galactopyranose (α-d-Gal*p*) were also observed at δ_DSS_ 4.51, 4.49, and 5.21 ppm. These values are consistent with those reported^36,37,49^ for RAG.

The ^13^C NMR spectrum of RAG (Figure 1D) shows the characteristic chemical shifts of the anomeric carbons of the sugars present in the polysaccharide structure, ranging from 98 to 115 ppm relative to the DSS reference standard. The peak at δ_DSS_ 112.1 ppm is assigned to the anomeric carbon of α-l-Ara*f*(1→, at δ_DSS_ 111.2 ppm to →3)-α-l-Ara*f*(1→, at δ_DSS_ 106.3 ppm to →3,6)-β-d-Gal*p*(1→, at δ_DSS_ 106.1 ppm to →3)-β-d-Gal*p*(1→, at δ_DSS_ 105.9 ppm to →6)-β-d-Gal*p*(1→, at δ_DSS_ 105.5 ppm to β-d-GlcA, at δ_DSS_ 104.2 ppm to α-l-Ara*p*(1→, at δ_DSS_ 103.3 ppm to Rha*p*(1→, at δ_DSS_ 102.1 ppm to β-l-Ara*p*(1→, and at δ_DSS_ 101.2 ppm to α-d-Gal*p*(1→. The signal at δ_DSS_ 178.0 ppm corresponds to the carbonyl carbon of β-d-GlcA, and the signal at δ_DSS_ 19.2 ppm is attributed to the methyl group (CH_3_) of Rha*p*. A similar ^13^C NMR spectrum for RAG has been reported by Chaves et al.^49^

The ^1^H–^13^C HSQC spectrum (Figure 1E) shows two anomeric carbon regions with high intensity signals, the first due to α-l-Ara*f* at different positions in the chain with ^1^H–^13^C correlations at δ_DSS_ 112.1/5.26, 111.3/5.38, and 110.9/5.43. The second region is due to β-l-Ara*p* linked to side chains assigned to δ_DSS_ 99.7/5.46, 99.7/5.51, 99.7/5.15, 99.3/5.43, and 99.3/5.22. The correlation at δ_DSS_ 104.1/5.09 was attributed to α-l-Ara*p*. Other correlations attributed to β-d-Gal*p*, β-d-GlcA, and α-d-Gal*p* were also observed at δ_DSS_ 105.9/4.51, 105.5/4.49, and 101.0/5.28, respectively.^36,37,50^ The configuration of the unusual α-d-Gal*p* unit was confirmed by gated decoupling ^13^C experiments, where a ^1^ *J*_C,H_ of 168.2 Hz was found, compatible with the anomeric configuration α in aldopyranoses (∼170 Hz);^51^ as no other aldopyranose was detected by HPLC, we believe that this residue should be a terminal α-d-Gal*p* residue as previously observed.^36,37^ The HSQC spectrum of partial hydrolyzed RAG (HP-RAG) ( Supporting Information, Figure S1) shows only two anomeric carbon signals with ^1^H–^13^C correlations at δ_DSS_ 106.1/4.43 and 106.4/4.68, correlations attributed to →3)-β-d-Gal*p*(1→ and β-d-Gal*p* terminals, respectively.

### 3.2 RAG Did Not Show *In Vitro* Cytotoxicity

RAG exhibited no cytotoxic effects against tumor and nontumor cell lines *in vitro* even at the highest tested concentration of 1000 μg/mL (Figure 2). Similar to our results, Dong et al.^52^ evaluated two arabinogalactan polysaccharides at concentrations of up to 1000 μg/mL and found no cytotoxicity on RAW264.7 macrophages or inhibition of cancer cell proliferation. In the study by Ribeiro et al.,^38^ quaternized and *in natura* RAG also did not show cytotoxic effects at a concentration of 500 mg/mL for nontumor cell lines L929 and HEK293T. Studies have shown that some polysaccharides do not present *in vitro* cytotoxic activity^10,52^ and others may be biologically active against tumor cells both *in vitro* and *in vivo.* ^53–55^ The biological activity presented by polysaccharides is influenced by the monosaccharide composition, molecular weight, and physicochemical factors.^56^

**Figure 2:**
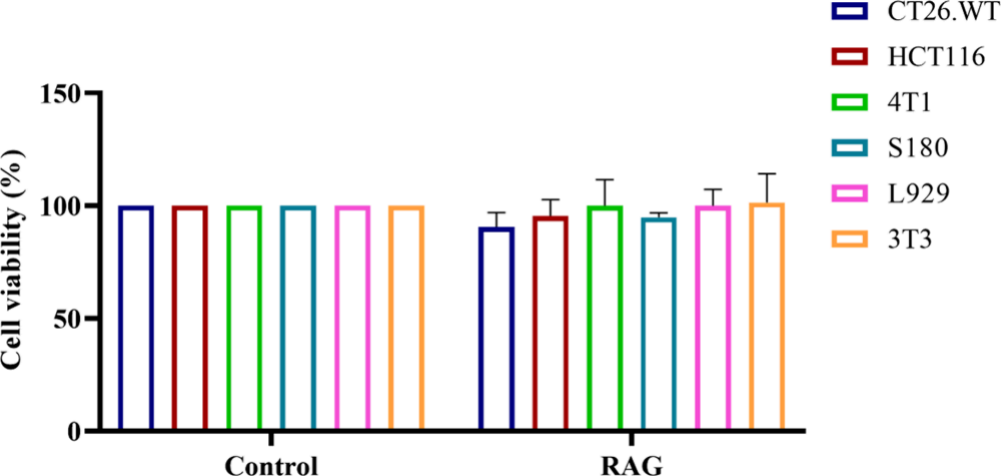
Cell viability (%) after 72 h of treatment with RAG (1000 μg/mL) in tumor cell lines CT26.WT (murine colorectal carcinoma), HCT116 (human colorectal carcinoma), 4T1 (murine mammary carcinoma), and S180 (sarcoma 180) and nontumor cell lines L929 and 3T3 (murine fibroblasts). Viability was measured using the MTT assay for all cell lines except S180, which was assessed with the Alamar Blue assay. All experiments were performed in duplicate.

### 3.3 RAG Exhibited Antitumor Effects *In Vivo*

RAG inhibited tumor growth in an S180 tumor-bearing mouse model. Both administered doses of 50 and 100 mg/kg/day of RAG inhibited 46.5 ± 14.4% (*p* < 0.05) and 36.6 ± 10.3%, respectively, of the tumor mass when compared with the group treated with saline solution. The use of the conventional chemotherapy drug 5-FU (15 mg/kg/day), used as a positive control in the experiment, showed a percentage of tumor inhibition of 60.8 ± 5.7% compared to the negative control (Figure 3A). These results demonstrate that the inhibitory effects of RAG on tumor growth are not dose-dependent since doubling the dose did not show greater efficacy. This suggests that the dose of 50 mg/kg/day achieves the maximum antitumor activity. Similar results were reported by Chen et al.^57^ in which polysaccharide isolated from *Cordyceps sinensis* showed a better antitumor effect at the lowest dose (100 mg/kg) than at the highest dose (200 mg/kg). The concentration–response relationship describes the important pharmacodynamic connection between the concentration of a drug and its biological effect. The classical assumption about the concentration–response relationship is that a drug is ineffective at low concentrations, shows moderate efficacy at intermediate concentrations, and reaches peak efficacy at higher concentrations, providing a sigmoidal curve. However, some drugs and reagents do not exhibit the classical concentration–response correlation and instead show bell-shaped or U-shaped curves, which are nonsigmoidal and have a nonmonotonic dose response. Thus, the relationship between the dose and effect is nonlinear, and higher doses may lead to saturation of a drug’s mechanisms of action or even to adverse effects that offset its efficacy.^58^

**Figure 3:**
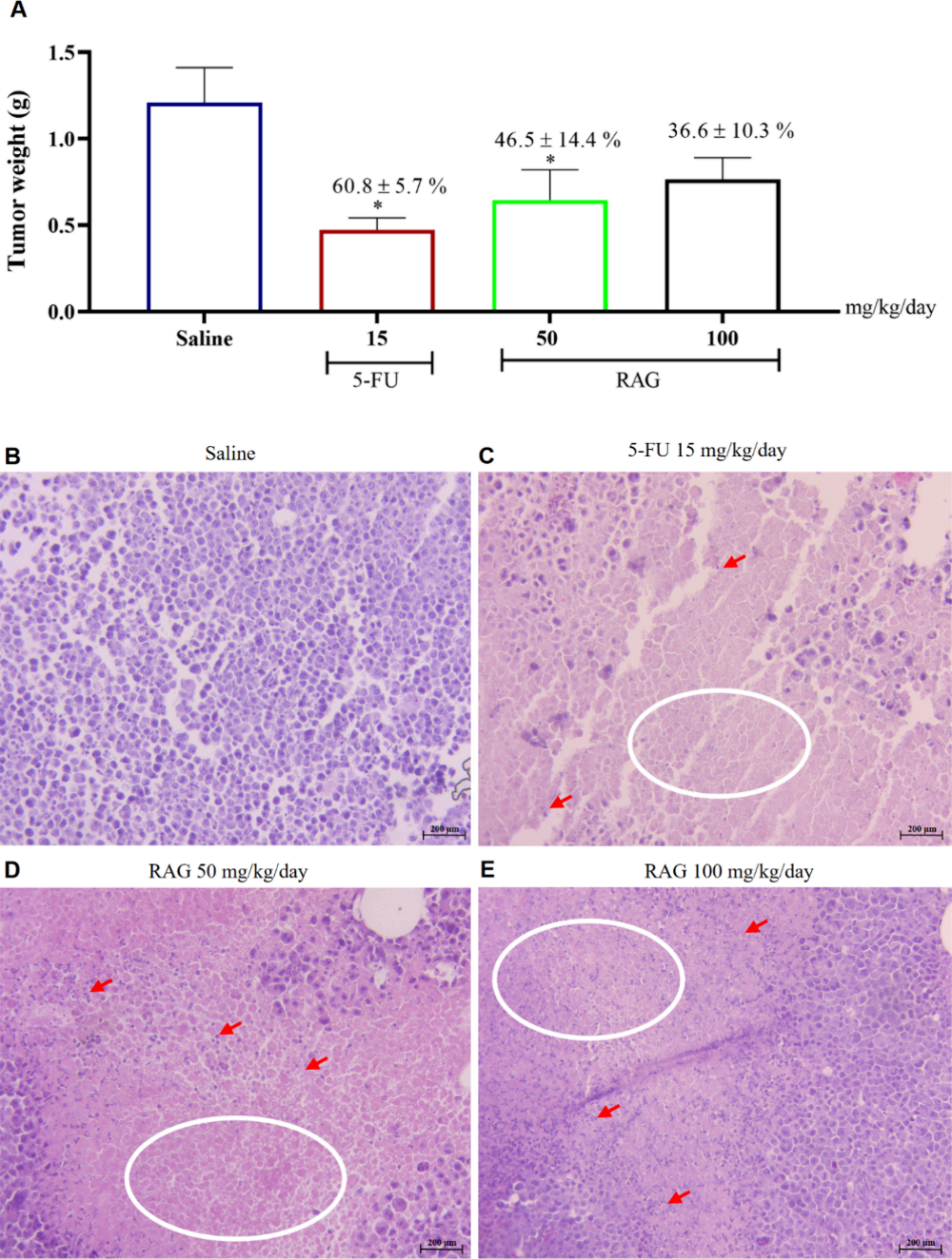
Tumor growth inhibition (A) and histopathological analysis (B–E) in S180 tumor-bearing mice treated with RAG (50 and 100 mg/kg/day). The negative control group received saline solution, and the positive control group received 5-fluorouracil (5-FU, 15 mg/kg/day). (A) Tumor volume data are presented as means ± SEM (*n* = 8 animals/group). *p* < 0.05 compared to the saline-treated control group (negative control), analyzed by ANOVA followed by Dunnett’s test. (B–E) Histological sections of tumor tissues (200× magnification). White circles = necrosis; red arrows = cellular debris. Tissues were stained with H&E and observed under a light microscope.

Histological sections of the tumor mass (Figure 3B–E) showed many cells with intense proliferative activity in the group treated with saline. However, the animals treated with RAG at doses of 50 and 100 mg/kg and 5-FU presented a smaller number of tumor cells and an area corresponding to necrosis. According to the literature, antitumor properties of polysaccharides can be mediated mainly by three approaches: direct cytotoxicity, modulation of the immune response, and through effects synergistic in combined treatment with conventional antitumor drugs.^15,59^

Given that RAG did not exhibit direct cytotoxic effects *in vitro*, the antitumor activity observed *in vivo* is likely mediated by indirect mechanisms, particularly through immunomodulatory pathways. Previous studies have shown that polysaccharides can enhance antitumor immunity by stimulating macrophage and dendritic cell phagocytosis, increasing natural killer (NK) cell cytotoxicity, activating B and T lymphocytes, and promoting the secretion of proinflammatory cytokines that contribute to tumor suppression.^60,61^ Several reports have specifically described arabinogalactans as potent immunomodulators capable of indirectly exerting antitumor effects.^15,62–65^ Therefore, it is plausible that the antitumor activity observed for RAG arises from its ability to modulate the immune system rather than from direct cytotoxicity. Nevertheless, additional studies are warranted to elucidate the precise molecular mechanisms underlying this therapeutic potential.

Previously, Moretão et al.^64^ showed that murine macrophages treated with white angico gum were stimulated to phagocytose S180 cells *in vitro*. In addition, ascitic sarcoma-bearing mice were submitted to i.p. treatment with white angico gum at doses of 50 and 100 mg/kg for 3 consecutive days and inhibition of cell growth was observed in 33 and 63%, respectively. These results led them to evaluate the antitumor potential of white angico gum at the highest dose (100 mg/kg) in an experimental model of S180 induced subcutaneously in mice, with a 38% inhibition of tumor growth being observed. These results corroborate ours, where RAG also showed the same percentage of tumor inhibition at a dose of 100 mg/kg/day.

Consistent with our findings, the arabinogalactan isolated from *Echinacea purpurea* exhibited no direct cytotoxicity *in vitro* against various cell lines but effectively suppressed tumor growth *in vivo* at a dose of 40 mg/kg in murine models of liver carcinoma (H22) and chemically induced murine colorectal cancer. This effect was attributed to macrophage activation toward the M1 phenotype, known for its proinflammatory and antitumor properties.^63^ Similarly, arabinogalactans obtained from *Anoectochilus formosanus* and *Oryza sativa* significantly inhibited the growth of murine colon tumors (CT26) at a dose of 15 mg/kg. The antitumor effects of these polysaccharides were primarily associated with enhanced immune modulation, including increased cytotoxicity of NK cells and T lymphocytes.^15,62^ Taken together, these comparisons highlight that, despite structural variability, arabinogalactans commonly exert indirect antitumor effects through immunomodulatory mechanisms. Within this context, RAG demonstrates comparable efficacy and safety, reinforcing its potential as a promising candidate among arabinogalactan-based biopolymers for anticancer therapy.

Although not statistically significant, RAG at the lowest dose (50 mg/kg/day) showed a slight trend toward higher tumor inhibition than the highest dose (100 mg/kg/day). This phenomenon may be related to receptor saturation or to the interaction of RAG with multiple targets, exhibiting counterbalancing effects at higher concentrations. Thus, bell-shaped dose–response curves at doses beyond the optimal range may reduce the biological response due to receptor saturation and lead to negative regulation of signaling pathways.^58^ Comparable results were reported by Araújo et al.,^21^ in which the RAG was evaluated in the antidiarrheal model at doses of 30, 60, and 120 mg/kg. Their findings revealed that among the tested doses, 60 mg/kg presented a significant reduction in the accumulation of intestinal fluids in a similar way to the highest dose, which occurred in the same way in our results.

### 3.4 RAG Did Not Produce Systemic Changes *In Vivo*

Regarding hematological parameters, RAG markedly altered only the hemoglobin concentration, which showed a slight significant decrease at the dose of 50 mg/kg/day in relation to the saline group. On the other hand, in addition to altering hemoglobin, 5-FU caused significant reductions in the number of total leukocytes and in the number of platelets, causing leukopenia and thrombocytopenia, aspects that were preserved in the groups treated with RAG, showing greater safety in its use (Figure 4A). It is important to highlight that no significant alterations were observed in other hematological parameters, organ weights, or serum biochemical markers. This indicates that the isolated decrease in hemoglobin at 50 mg/kg is not biologically relevant and does not compromise the safety profile of RAG. Such a finding may reflect physiological adaptation rather than toxic response, as reported for other natural polysaccharides. These results further support that, despite its antitumor efficacy, RAG exhibits a high safety margin when compared to 5-FU.

**Figure 4:**
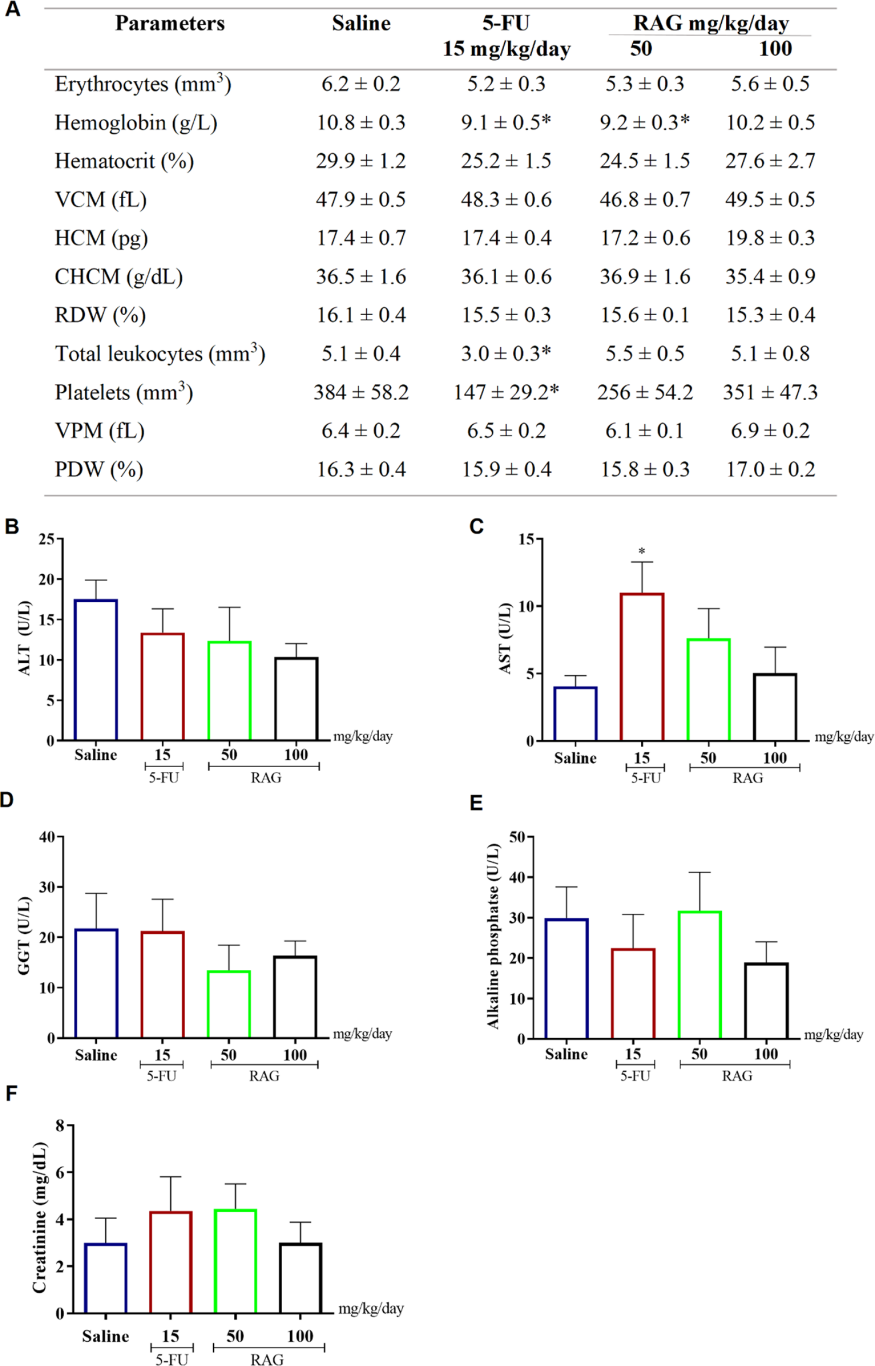
Hematological (A) and biochemical (B–F) parameters of mice treated with RAG (50 and 100 mg/kg/day i.p.), after 10 days of treatment. The negative control group received saline solution, and the positive control group received 5-fluorouracil (5-FU, 15 mg/kg/day). Values correspond to the mean ± SEM (*n* = 8 animals/group). *p < 0.05 compared to the group treated with saline solution (negative control) by ANOVA followed by Dunnett’s test.

The analysis of the serum biochemical profile (Figure 4B–F) generally revealed that RAG did not induce significant changes when compared to the saline group. However, the positive control (5-FU) significantly increased AST parameters compared to the negative control.

The body’s response to toxic substances can be manifested by physiological changes and cellular damage to the body.^44,47^ The absence of systemic toxicity shown in our results was also observed for two arabinogalactans, lemon gum^56^ and cashew gum.^10^

### 3.5 RAG Did Not Induce Signs of Toxicity

Analysis of body weight, survival, and relative weight of the excised organs (spleen, heart, liver, and kidneys) (Table 1) showed no significant difference compared to the saline-treated group, indicating the absence of toxicity by RAG at both doses. Likewise,^8,10,21,56^ others did not observe signs of *in vivo* toxicity with the use of natural polysaccharides in their respective studies.

**Table 1:** Effect of Treatments with Saline Solution, 5-FU (15 mg/kg/day), and RAG (50 and 100 mg/kg/day) on the Initial and Final Body Weight, Survival, and Relative Organ Weight of Mice Bearing Sarcoma 180^a^

		5-FU	RAG (mg/kg/day)	
parameters	saline	15 mg/kg/day	50	100
survival start/end of experiment	8/8	8/8	8/8	8/8
initial body weight (g)	32.5 ± 1.8	31.4 ± 1.2	33.1 ± 1.2	31.8 ± 1.3
final body weight (g)	29.4 ± 1.6	28.2 ± 1.1	30.3 ± 1.2	29.8 ± 1.2
heart (g/100 g of body weight)	0.5 ± 0.02	0.4 ± 0.01	0.6 ± 0.03	0.4 ± 0.02
kidney (g/100 g of body weight)	1.1 ± 0.05	1.0 ± 0.05	1.0 ± 0.02	1.1 ± 0.05
liver (g/100 g of body weight)	5.0 ± 0.3	4.0 ± 0.2*	4.9 ± 0.1	4.5 ± 0.2
spleen (g/100 g of body weight)	0.7 ± 0.05	0.3 ± 0.03*	0.6 ± 0.03	0.6 ± 0.03

a Values correspond to the mean ± SEM (*n* = 8 animals/group). *p < 0.05 compared to saline group, analyzed by ANOVA followed by Student–Newman–Keuls.

Most polysaccharides are described as relatively nontoxic and do not cause significant side effects, making them great candidates for therapeutic applications.^66^ Nevertheless, the relative weight of the organs in the experimental group treated with the standard chemotherapy drug 5-FU differs in terms of the relative weight of the spleen and liver, these immune and metabolic organs demonstrating a decrease in their weights compared to the saline group (Table 1). 5-Fluoruracil and its metabolites are toxic to normal cells, leading to serious adverse reactions in the body that manifest mainly as damage to the immune system and some organs such as the liver, kidneys, and small intestine.^42,67,68^

The histopathological analysis corroborates the previous results, as no evidence of toxicity or histopathological changes was observed by RAG at any dose tested. In the heart sections, the images of the group treated with the polymer showed healthy cardiac tissues like the saline group, as a normal conformation of the muscle fibers can be observed (black arrows). The analysis of the kidneys treated with RAG showed a normal glomerulus (G), with Bowman’s space and well-regulated distal and proximal convoluted tubes, characteristics similar to the negative control group. The liver section of animals treated with RAG showed the central vein (VC) and hepatocytes without histological changes compared to the saline group. In the spleen, both the treated groups and the saline group present the usual architecture such as red pulp and white pulp (red and yellow dashes) (Figure 5).

**Figure 5:**
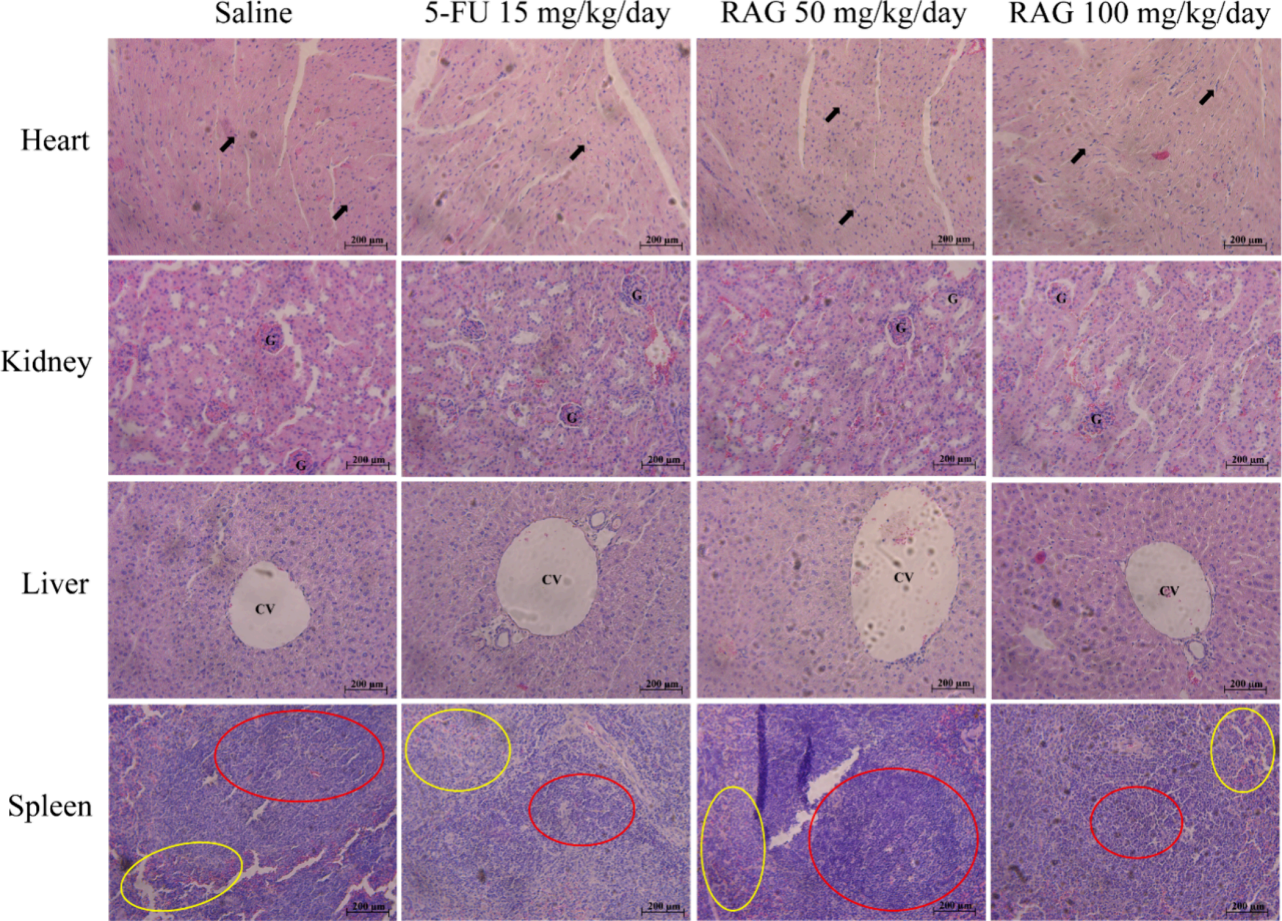
Histopathological evaluation of the heart, kidneys, liver, and spleen of animals treated with RAG (50 and 100 mg/kg/day), 5-FU (15 mg/kg/day), and saline for 10 days consecutive (200×). Black arrows = muscle fibers; G = glomerulus; CV = central vein; red and yellow dashed lines outline the red and white pulp, respectively.

Other studies showed similar results with the use of gum arabic,^69^ gum tragacanth,^70^ cashew gum,^10,71^ lemon gum,^56^ and RAG via the oral route.^38^ The benefits of polysaccharides are on the rise due to their diverse bioactivities and low or absence of toxicity and protective effects, which differentiates them from current antineoplastic agents.

### 3.6 RAG Did Not Induce *In Vivo* Mutagenicity

Bone marrow cells from animals that received daily treatment with RAG at both doses were analyzed to evaluate the safety of its use and its potential genotoxicity. Thus, 8000 polychromatic erythrocytes from the bone marrow of each group were analyzed and it was demonstrated that RAG did not cause micronucleus formation at any administered dose compared to the saline group. In contrast, 5-FU was potentially toxic to the genetic material, causing the micronucleus formation in treated animals when compared to the negative control. The EPC/NCE ratio was considered without significant changes by RAG, unlike 5-FU, which had a significant decrease compared to the group treated with saline (Table 2).

**Table 2:** Evaluation of the Mutagenic Effect in Bone Marrow Cells of Mice Treated with RAG (50 and 100 mg/kg/day), 5-FU (15 mg/kg/day), and Saline for 10 Consecutive Days^a^

group (mg/kg/day)	EPC analyzed (*n*)	EPCMN	EPC/ENC
saline	8000	5.1 ± 1.08	1.37 ± 0.09
5-FU 15	8000	37.2 ± 2.8*	1.11 ± 0.07*
RAG 50	8000	7.4 ± 1.7	1.33 ± 0.06
RAG 100	8000	9.1 ± 2.1	1.29 ± 0.09

a *p < 0.05 compared to the saline group, analyzed by Student’s *t* test (*n* = 8 animals/group). EPC: polychromatic erythrocytes; EPCMN: polychromatic erythrocytes with micronucleus; ENC: monochromatic erythrocytes.

The micronucleus assay is a widely used tool to assess the exposure of organisms to chemical or physical agents, being an important assay in genetic toxicology and in the evaluation of the carcinogenic potential of bioactive pharmacologically promising substances.^72^ It is essential to discover substances that are safe and nontoxic to combat various diseases, including cancer.^73^ Our results demonstrate the safety of daily i.p. administration of RAG. Similar to our results, Oliveira et al.^72^ observed that a β-glucan polysaccharide isolated from *Saccharomyces cerevisiae*, composed of d-glucose linked at the β-(1→3) position with β-(1→6) side chains, did not cause changes in the micronucleus frequency at different doses in peripheral blood cells of mice.

### 3.7 RAG Presented Antiedematogenic Effects *In Vivo*

At a dose of 50 mg/kg, RAG significantly decreased paw edema induced in the animals at all four time points (1, 2, 3, and 4 h) by 21.7, 27.8, 19.1, and 5.6%, respectively, compared to the saline-treated group. The 100 mg/kg dose showed a decrease in edema in the first 3 h, corresponding to 21.7, 18.2, and 14.3%, demonstrating that the 50 mg/kg dose of RAG was effective for a longer period. The positive control used a standard drug indomethacin at 10 mg/kg, which showed a decrease in edema in the four evaluated times corresponding to 26, 40.9, 47.6, and 38.8, when compared to the group treated only with saline (Figure 6).

**Figure 6:**
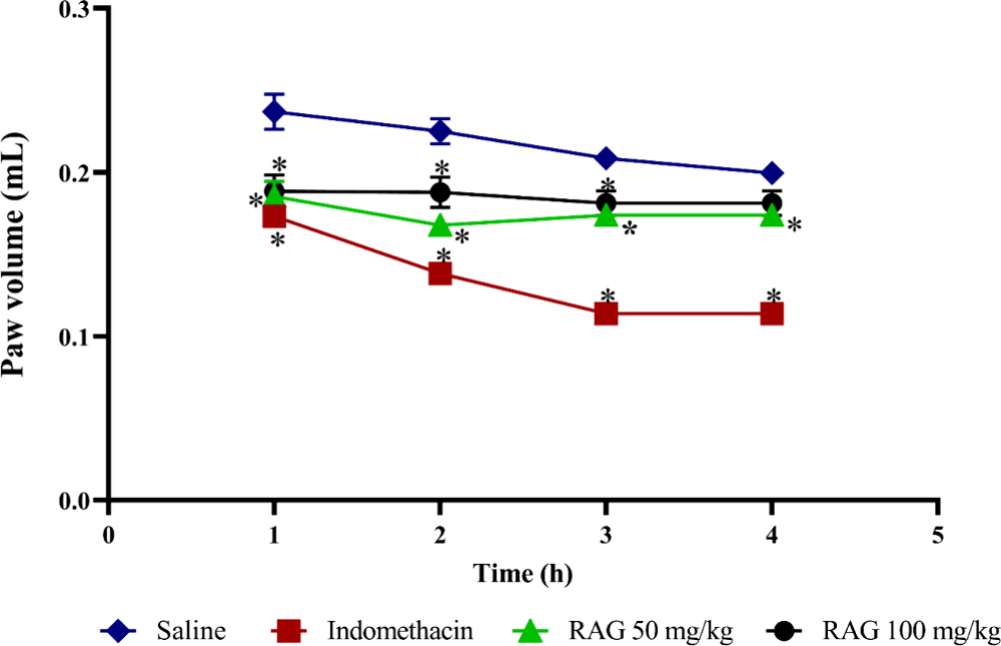
Antiedematogenic effect in mice pretreated intraperitoneally with RAG at doses of 50 and 100 mg/kg four times (1, 2, 3, and 4 h). Values correspond to the mean ± SEM (*n* = 8 animals/group). *p < 0.05 compared to the group treated with saline solution (negative control) by ANOVA followed by Dunnett’s test.

In recent years, natural products including polysaccharides, terpenoids, flavonoids, polyphenols, and others have demonstrated promising anti-inflammatory effects both *in vitro* and *in vivo*.^74,75^ Maria-Ferreira et al.^76^ and Carlotto et al.^77^ evaluated polysaccharides from the arabinogalactan class in paw edema induced by carrageenan in mice, and their respective results demonstrated a significant decrease in edema with treatment by isolated polysaccharides.

Polysaccharides involve several mechanisms of action to control inflammation. For example, inhibiting the release of inflammatory mediators or regulating proinflammatory and anti-inflammatory cytokines is an excellent strategy used to control the inflammatory process. In addition, polysaccharides promote the suppression of inducible nitric oxide synthase (iNOS) and cyclooxygenase (COX) as a strategy since these massively stimulate the inflammatory process. Nuclear factor kappa B (NF-kB) is one of the most important effector pathways involved in inflammation; when activated, it generates a cascade of genes that lead to the production of various mediators; in this sense, many polysaccharides suppress the activation of this pathway as an underlying mechanism. In addition, it is worth noting that natural polysaccharides can have such effects through regulation of the immune system, activation of signal transducers and transcription activators 3 (STAT 3), or histamine blockade.^74^ Given the many mechanisms of action employed by polysaccharides, our study demonstrated an antiedematogenic effect by RAG at the two tested doses.

## 4 Conclusions

RAG is a polysaccharide composed mainly of arabinose and galactose with smaller proportions of rhamnose and glucuronic acid. This polymer with few protein traces presented a type II arabinogalactan profile. It was concluded that RAG did not show *in vitro* cytotoxic effects for tumor and nontumor cell lines; however, it was shown to have antitumor potential in an *in vivo* model against S180 with no systemic and tissue changes. The *in vivo* administration of the polymer revealed no mutagenic effects, presenting greater biocompatibility and safety, unlike the chemotherapy drug 5-FU. Furthermore, an antiedematogenic effect was observed, suggesting a potential anti-inflammatory activity. These are the first results presented regarding the antitumor and antiedematogenic potential exerted by RAG, and further studies are needed to explore the mechanistic processes that this polymer provides.

## Supplementary Material



## Notes

(Figure S1) HSQC spectrum of partial hydrolyzed RAG (PDF)
