# Supplementary material for: Red Angico Gum: A Nontoxic Polysaccharide with Promising Antitumor and Antiedematogenic Activity
Source: ACS Omega. 2025 Nov 13;10(46):56656–69. doi: 10.1021/acsomega.5c09191 (PMC12658711; doi:10.1021/acsomega.5c09191)
Supplement: Supplementary file 1 [file ao5c09191_si_001.pdf]

Supplementary Material

Red Angico Gum: A Non-Toxic Polysaccharide with Promising Antitumor and Antiedematogenic Activity

*Dakson Douglas Araújo[1], Rayran Walter Ramos de Sousa[2], Débora Caroline do Nascimento Rodrigues[2], Ingredy Lopes dos Santos[2], Paulo Michel Pinheiro Ferreira[2], Antonielly Campinho dos Reis[3], Maria Luísa Lima Barreto do Nascimento[3], João Marcelo de Castro e Sousa[3], Fábio de Oliveira Silva Ribeiro[4], Durcilene Alves da Silva[4], Irisvan Silva Ribeiro[5], Venicios Gonçalves Sombra[5], Regina Célia Monteiro de Paula[5], Nayze Lucena Sangreman Aldeman[6,7], José Delano Barreto Marinho-Filho[1], Ana Jérsia Araújo[1],**

[1] *Laboratório de Cultura de Células do Delta (LCCDelta), Universidade Federal do Delta do Parnaíba, Parnaíba, PI, Brazil.*

[2] *Laboratório de Cancerologia Experimental (LabCancer), Departamento de Biofísica e Fisiologia, Universidade Federal do Piauí, Teresina, PI, Brazil.*

[3] *Laboratório de Genética Toxicológica (LAPGENIC), Departamento de Bioquímica e Farmacologia, Universidade Federal do Piauí, Teresina, Teresina, PI, Brazil.*

[4] *Núcleo de Pesquisa em Biodiversidade e Biotecnologia (BIOTEC), Universidade Federal do Delta do Parnaíba, Parnaíba, PI, Brazil.*

[5] *Laboratório de Polímeros (LabPol), Universidade Federal do Ceará, Fortaleza, CE, Brazil.*

[6] *Curso de Medicina, Universidade Federal do Delta do Parnaíba, Parnaíba, PI, Brazil.*

[7] *Faculdade de Ciências Humanas, Exatas e da Saúde do Piauí, Instituto de Educação Superior do Vale do Parnaíba, Parnaíba, PI, Brazil.*

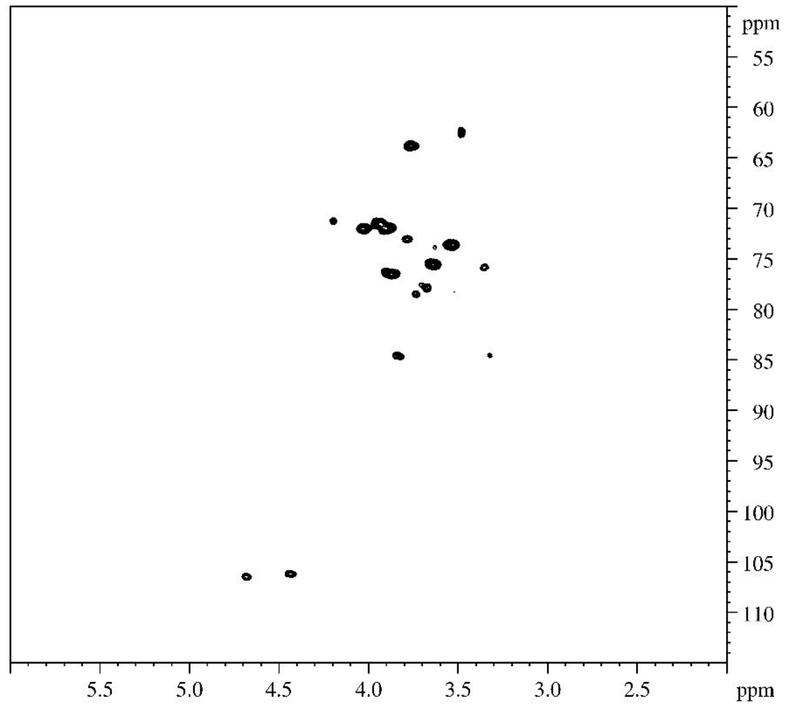

Figure S1 1H-13C HSQC of partial hydrolysed RAG (HP-RAG)
